# Editorial: Gluten-related disorders: pathogenesis, diagnosis, and treatment

**DOI:** 10.3389/fnut.2025.1585381

**Published:** 2025-03-13

**Authors:** Simone Baldi, Ángela Ruiz-Carnicer, Isabel Comino, Carolina Sousa, Amedeo Amedei

**Affiliations:** ^1^Department of Experimental and Clinical Medicine, University of Florence, Florence, Italy; ^2^Department of Microbiology and Parasitology, Faculty of Pharmacy, University of Seville, Seville, Spain

**Keywords:** gluten related disorders, celiac disease, gluten free diet (GFD), celiac disease—diagnosis, diet therapy, epidemiology

Celiac disease (CD) is a systemic autoimmune disorder triggered by gluten peptides, which provoke a T-cell-mediated immune response, leading to villous atrophy and chronic inflammation in the small intestine (1, 2). It affects ~1.4% of the global population (3), with a highly variable clinical presentation that ranges from classic gastrointestinal symptoms to extraintestinal manifestations or even asymptomatic cases. The only effective treatment is lifelong adherence to a gluten-free diet (GFD) (4). Recent studies suggest that the loss of gluten tolerance may occur either upon initial gluten exposure or later in life, implying that additional environmental factors contribute to CD development. Challenges in diagnosis and poor adherence to the GFD can significantly impact health and quality of life, highlighting the need for early and accurate diagnosis to improve long-term disease management (5).

This Research Topic brings together nine comprehensive articles exploring the pathogenesis, diagnosis, and management of gluten-related disorders, aiming to deepen our understanding of these crucial aspects.

CD diagnosis relies on clinical presentation, serological markers, and histopathological findings from duodenal biopsies. However, discrepancies between serological and histological results, along with biopsy inaccessibility, often lead to inconclusive outcomes. Since most CD patients carry the HLA-DQ2 and/or DQ8 alleles, which trigger an autoimmune response, HLA testing serves as a valuable complementary tool, particularly in atypical or controversial cases where histological findings are inconclusive (Ruera et al.). Atypical CD presentations may include constitutional symptoms, dermatological and mucosal issues, bone abnormalities, neuropsychiatric symptoms, renal and reproductive complications, disturbances in biological markers, and associations with other autoimmune conditions. Recognizing this diverse clinical spectrum is crucial for optimal patient management, especially in ensuring proper growth and development in children (Lupu et al.). Given the heterogeneous presentation of CD, a multifaceted diagnostic approach is essential. Early and accurate diagnosis improves treatment effectiveness and quality of life, particularly for patients at risk of poor adherence due to unclear or delayed diagnoses.

Emerging research has identified gluten immunogenic peptides (GIP) in human breast milk, with significant interindividual variations in secretion. This discovery is particularly relevant, as early gluten exposure through breast milk may influence the immune system's development in genetically predisposed infants, potentially affecting CD risk. Understanding this mechanism is crucial for identifying early-life environmental factors that contribute to CD onset, providing insights for more effective prevention strategies (Ruiz-Carnicer et al.).

Additionally, GIP detection in urine offers a non-invasive method for assessing gluten exposure and gastrointestinal function following dietary challenges or fasting. This approach is particularly useful for identifying subclinical CD cases and monitoring treatment effectiveness. By tracking GIP levels in urine, it becomes possible to detect gluten ingestion in patients who may not exhibit obvious symptoms, enabling earlier diagnosis and better management. These findings, together with GIP detection in breast milk, emphasize the role of early gluten exposure in CD development and its potential for precise disease monitoring (Rodríguez-Ramírez et al.).

Regarding CD treatment, a strict lifelong GFD remains the cornerstone; however, adherence is often challenging due to high costs, dietary restrictions and social implications. Recent phase 2 clinical trials exploring non-dietary pharmacological therapies for CD underscore the need for more extensive research, as no proposed treatments have yet shown significant efficacy in preventing gluten-induced histological damage (Scalvini et al.). Hence, larger and more rigorous clinical trials are necessary to assess their long-term safety and effectiveness. A promising therapeutic approach involves exopeptidases, enzymes that break down gluten-derived peptides in the gastrointestinal tract, potentially reducing the immune response and alleviating CD symptoms. Although still under investigation, this strategy holds potential for improving current treatment options and enhancing quality of life for CD patients (Mourabit et al.).

The development of gluten-free food products, such as cookies, is being evaluated for their physicochemical and sensory properties ensuring that CD patients have access to safe, nutritious and palatable food options (Silva-Paz et al.). The availability of high-quality gluten-free foods plays a crucial role in promoting dietary adherence and improving patient's overall experience with GFD.

Beyond dietary restrictions, the psychosocial burden of CD is becoming increasingly apparent. The need to evaluate health-related quality of life (HRQoL) in CD patients is critical, as dietary limitations often contribute to social isolation, anxiety and psychological distress. These challenges are further exacerbated by diagnostic difficulties and the constant need to avoid gluten exposure.

Recent research comparing general and disease-specific HRQoL questionnaires have provided deeper insights into the psychosocial impact of CD (Falcomer et al.). A study conducted in Portugal highlighted the necessity of a comprehensive care model integrating both medical and psychosocial support. Such a holistic approach is essential for enhancing patient outcomes and ensuring that individuals with CD can maintain a fulfilling quality of life despite the challenges of their condition (Chaves et al.).

In conclusion, significant progress has been made in understanding the pathogenesis, diagnosis, and treatment of gluten-related disorders. While the GFD remains the primary treatment, emerging pharmacological therapies, such as exopeptidase-based approaches, offer potential alternatives for individuals struggling with gluten avoidance. Additionally, increasing awareness of the psychosocial impact of CD underscores the importance of a multidisciplinary care approach that addresses both physical and emotional wellbeing. Looking ahead, effective management of gluten-related disorders will require precise diagnostic tools, innovative therapies and a patient-centered, integrative approach to optimize clinical care and enhance quality of life.
